# Human-like PB2 627K Influenza Virus Polymerase Activity Is Regulated by Importin-α1 and -α7

**DOI:** 10.1371/journal.ppat.1002488

**Published:** 2012-01-19

**Authors:** Ben Hudjetz, Gülsah Gabriel

**Affiliations:** Heinrich-Pette-Institute, Leibniz Institute for Experimental Virology, Hamburg, Germany; University of Wisconsin-Madison, United States of America

## Abstract

Influenza A viruses may cross species barriers and transmit to humans with the potential to cause pandemics. Interplay of human- (PB2 627K) and avian-like (PB2 627E) influenza polymerase complexes with unknown host factors have been postulated to play a key role in interspecies transmission. Here, we have identified human importin-α isoforms (α1 and α7) as positive regulators of human- but not avian-like polymerase activity. Human-like polymerase activity correlated with efficient recruitment of α1 and α7 to viral ribonucleoprotein complexes (vRNPs) without affecting subcellular localization. We also observed that human-like influenza virus growth was impaired in α1 and α7 downregulated human lung cells. Mice lacking α7 were less susceptible to human- but not avian-like influenza virus infection. Thus, α1 and α7 are positive regulators of human-like polymerase activity and pathogenicity beyond their role in nuclear transport.

## Author Summary

Adaptive mutations in the polymerase complex, such as PB2 E627K and PB2 D701N play a crucial role in influenza virus interspecies transmission. We have shown earlier that PB2 D701N promotes nuclear entry of PB2 by enhanced importin-α binding in mammalian cells (Gabriel et al., PLoS Pathogens 2008; Gabriel et al., Nat. Commun. 2011). In this study, we show that the adaptive mutation PB2 E627K involves an interaction with importin-α by a novel mechanism that does not mediate nuclear transport of viral factors. We found that human importin-α1 and -α7 are required for efficient human- (PB2 627K) but not avian-like (PB2 627E) polymerase activity. Further, human-like polymerase activity correlated with enhanced importin-α1 and -α7 binding to viral ribonucleoprotein complexes (vRNP). Mice lacking importin-α7 were less susceptible to human-like but not avian-like influenza virus infection. Our findings strongly suggest that importin-α isoforms possess functions beyond their primary role in nuclear transport facilitating PB2 E627K mediated host adaptation and pathogenicity.

## Introduction

Influenza A viruses are able to cross species barriers and transmit to humans leading to disease of various severity. Interspecies transmission of influenza viruses is multigenic involving several viral and cellular factors. Host restriction occurs mainly by two cellular barriers which need to be overcome upon transmission. First, upon entry viruses need to cross the cell membrane by interaction of the viral receptor binding protein, the hemagglutinin (HA), to the adequate host cell receptors consisting of sialic acid-containing glycoproteins [1], [2]. Second, vRNP components (PB2, PB1, PA and NP), especially PB2 and NP need to adapt to the nuclear import machinery in order to establish efficient replication in the nucleus of the new host cell [3]–[5].

The adaptive position in the viral polymerase subunit PB2 at position 627 is a well known determinant of host range and pathogenicity [6]. Influenza viruses of avian origin are characterized by a glutamic acid signature at this position while human viruses predominantly possess a lysine signature [7]. The PB2 E627K mutation has been shown to increase viral polymerase activity [3] and pathogenicity in mammalian hosts [8], [9].

Despite intensive investigations the molecular basis underlying the host adaptive position 627 in PB2 is poorly understood. It has been shown, that polymerase complexes containing PB2 627E display a defect in vRNP complex assembly leading to restricted polymerase activity and impaired virus growth in mammalian cells [10], [11]. It has been postulated that an unknown cellular inhibitor specifically restricts avian-like polymerase activity in human cells [11]. On the other hand, it has also been proposed that there is no evidence for the existence of a mammalian inhibitory factor of avian-like polymerases but instead the absence or low expression of a positive factor is responsible for low avian polymerase activity in human cells [12]. Very recently, the DEAD box RNA helicase DDX17/p72 has been identified among other polymerase interacting proteins [13] to facilitate efficient H5N1 627K virus transcription and replication in human cells [14]. Consistent with the hypothesis that PB2 627K position affects polymerase-host interaction, it was shown that an E627K substitution alters the electrostatic surface potential of the 627-domain resulting in a basic patch, possibly modulating interactions between viral and host factors [15], [16]. However, cellular factors involved in PB2 627K mediated host adaptation and pathogenicity still remain poorly understood.

It was shown that cellular importin-α isoforms play an essential role in influenza virus host adaptation [4], [5]. Importin-α proteins are components of the classical import pathway and act as adaptors recognizing cargo proteins with a nuclear localization signal (NLS). Importin-α/cargo protein complexes facilitate binding to the importin-β1 receptor protein. Thus, cargo proteins are transported into the nucleus as ternary complexes [17], [18]. Adaptive mutations in PB2 D701N and NP N319K have been shown to be adaptations to cellular importins thereby allowing efficient nuclear transport of PB2 and NP and thus resulting in enhanced virus polymerase activity in mammalian cells [3], [4]. It has been recently shown that avian and mammalian influenza viruses possess differential preferences for importin-α isoforms in human lung cells [5]. While growth of highly pathogenic avian influenza (HPAIV) viruses with avian signatures (PB2 627E or PB2 701D) depended on importin-α3, viruses with mammalian signatures (PB2 627K or PB2 701N) depended on importin-α7. Thus, a switch from importin-α3 to importin-α7 dependency occurs upon mammalian adaptation. Analyzing the role of the PB2 701 polymorphism revealed that adaptive mutations in PB2 D701N and NP N319K mediate the switch from importin-α3 to importin-α7 dependency upon avian-mammalian transmission [5]. Whether other host adaptive positions, such as PB2 627K can mediate a switch to importin-α7 dependency similar to PB2 710N was not further investigated. However, functional substitutions between PB2 627K and 701N have been described before. Either position could compensate for the lack of the other position resulting in increased virus transmission in a guinea pig model [19].

We have initiated this study to analyze whether the host adaptive substitution in PB2 E627K is also involved in importin-α dependent host adaptation. Here, we investigated the role of cellular importins in the regulation of PB2 627K mediated influenza virus polymerase activity and pathogenicity.

We have identified human importin-α1 and -α7 as positive regulators of human- (PB2 627K) but not avian- (PB2 627E) like polymerase activity. In contrast, human importin-α3 acts as a general negative regulator of human- and avian-like polymerase activity in vRNP reconstitution assays while virus growth was not affected. Increased human-like polymerase activity correlated with efficient recruitment of importin-α1 and -α7 to vRNPs in human cells. In contrast, avian-like PB2 627E failed to recruit these importin-α isoforms efficiently to vRNPs. Interestingly, subcellular localization of PB2 627 mediated human- and avian-like vRNP components was not affected by importin-α proteins as reported before with PB2 D701N [4] suggesting a novel mechanism triggered by PB2 627K. Consistent with these findings, human-like PB2 627K but not avian-like PB2 627E virus growth depended on importin-α1 and -α7 in human lung cells and displayed reduced pathogenicity in importin-α7 knockout (α7^−/−^) mice. Our findings described here show that importin-α1 and -α7 act as positive regulators of human- (PB2 627K) but not avian- (PB2 627E) like polymerase activity without affecting nuclear transport of viral vRNPs. Thus, cellular importin-α proteins play an important role in PB2 627K mediated interspecies transmission beyond their primary role in nuclear transport.

## Results

### Human-like polymerase activity depends on importin-α1 and -α7 in human cells

We have previously shown that growth of avian and human influenza viruses depends on different importin-α isoforms [5]. Further, it has been proposed that avian-like polymerase complexes containing PB2 627E are specifically restricted by an unknown host factor which is only present in a mammalian host environment [11]. Moreover, the crystal structure of the PB2 domain containing 627K led to a basic patch on the protein surface upon PB2 E627K substitution [15]. Basic amino acids are classical binding motives for importin-α proteins such as found in NLS sequences of cargo proteins.

**Figure 1:**
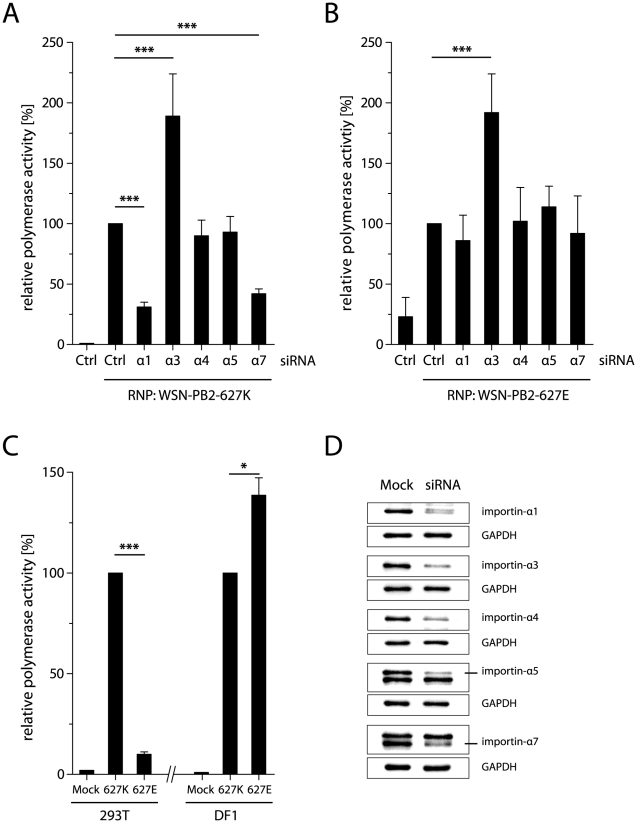
Importin-α1 and -α7 are required for human-like polymerase activity in human cells. (A and B) Polymerase activity of WSN-PB2 627K (A) or WSN-PB2 627E (B) containing vRNPs in importin-α siRNA silenced human 293T cells. Activity of vRNPs in negative siRNA silenced cells (Ctrl) was set 100%. As a background control, vRNPs were transfected omitting the PB2 subunit in negative siRNA silenced cells (Ctrl). Means of at least three independent experiments +/− standard deviations (SD) are shown (*p<0.05, **p<0.01, ***p<0.001, by students *t*-test). (C) Polymerase activity of WSN-PB2 627K or WSN-PB2 627E containing vRNPs in human 293T (left) and avian DF1 (right) cells. Mock transfected cells were used a control (Mock). (D) Confirmation of importin-α silencing in human 293T cells by Western blot analysis. GAPDH was used as a loading control.

Therefore, we addressed the question whether cellular importins are involved in the regulation of influenza virus polymerase activity mediated by the PB2 627K polymorphism. We performed a cell-based polymerase activity assay in combination with siRNA-mediated silencing of individual importin-α proteins. vRNPs of WSN containing either PB2 627K or PB2 627E were reconstituted in importin-α silenced 293T cells (Figure 1D) and their activity was compared to vRNPs expressed in control siRNA treated cells which was set 100% (Figure 1A and B). Silencing of importin-α1 and importin-α7 significantly decreased human-like PB2 627K polymerase activity to 31% and 42%, respectively, compared to negative siRNA controls (Figure 1A). In contrast, PB2 627K activity was significantly increased up to 189% in importin-α3 silenced cells. No significant effects were observed in importin-α4 or -α5 silenced cells. In contrast, silencing of either importin-α isoform (importin-α1, -α4, -α5 or -α7) except for importin-α3 did not affect avian-like PB2 627E polymerase activity (Figure 1B). Silencing of importin-α3 led to increased PB2 627E polymerase activity (192%) compared to control siRNA treated cells. However, avian-like polymerase activity was severely impaired in human cells with a 10-fold reduction (10%) compared to human-like polymerase activity (Figure 1C, left). On the other hand, avian-like polymerase activity was slightly increased (132%) in comparison to human-like polymerase activity in avian cells (Figure 1C, right). This further confirms that avian-like PB2 627E polymerase complexes are restricted in human but not in avian cells.

Here we show that importin-α1 and -α7 are required for efficient human-like but not avian-like polymerase activity in human cells. In contrast, human importin-α3 restricts both human and avian polymerase activities. These findings suggest that importin-α1 and -α7 are positive regulators of human-like polymerase activity while importin-α3 is a negative regulator of human- and avian-like polymerase activities.

### Human- and avian-like PB2 display similar human importin-α binding affinities

**Figure 2:**
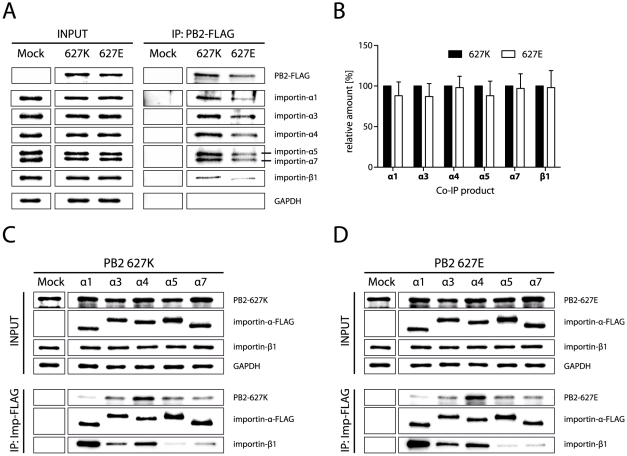
PB2 627K and PB2 627E monomers display similar importin-α binding affinities. (A) PB2 binding to endogenous importins. 293T cells were transfected with expression plasmids for PB2-627K-FLAG or PB2-627E-FLAG. Mock transfected cells served as a control (Mock). Cells were harvested 48 h after transfection and subjected to immunoprecipitation of FLAG-tagged PB2 proteins. The amount of co-immunoprecipitated endogenous importins (α1, α3, α4, α5, α7 and β1) was analyzed by Western blot. (B) Quantification of PB2 binding to endogenous importins in (A) using a Bioimager as described in Experimental Procedures. Precipitated amounts of importins were normalized against precipitated PB2 levels. Data shown are derived from at least three independent experiments and represent mean +/− SD. (C and D) PB2 binding to overexpressed importins. 293T cells were co-transfected with expression plasmids for FLAG-tagged importin-α proteins (α1, α3, α4, α5 or α7) and untagged PB2 627K (C) or PB2 627E (D). PB2-only transfected cells served as a control (Mock). Cells were lysed 48h after transfection and immunoprecipitated using the FLAG-tag. The amount of co-immunoprecipitated PB2 and importin-β1 was determined by Western blot analysis.

In order to understand the molecular basis for differential regulation of polymerase activities by importins, we assessed binding properties of PB2 627K-FLAG and PB2 627E-FLAG in human cells by immunoprecipitation analysis. As cargo proteins are transported as ternary complexes into the nucleus, in this case PB2/importin-α/importin-β1 [17], [18], we assessed the binding of PB2 to importin-α isoforms and detected importin-β_1_ levels precipitated from PB2/importin-α complexes as described before [4]. Both, avian- and human-like PB2 proteins were found to interact with endogenous importin-α isoforms as well as the corresponding importin-β_1_ receptor to similar affinities (Figure 2A and 2B). Co-immunoprecipitations studies with co-transfected importin-α-FLAG and PB2 627K or PB2 627E were carried out to investigate the importin-α binding properties to both PB2 proteins. Here, no significant differences have been observed in importin-α or importin-β_1_ binding affinities to PB2 627K or PB2 627E. Both PB2 variants showed highest binding affinity to importin-α4. Importin-α5 and -α7 displayed lowest affinity to the importin-β_1_ receptor protein (Figure 2C and 2D).

In summary, we show that human- as well as avian-like PB2 proteins possess similar binding affinities to their adaptor importin-α and receptor importin-β_1_ complexes. These findings suggest that PB2 E627K substitution does not alter importin-α binding or result in differential importin-α/importin-β_1_ binding properties suggestive of altered nuclear transport.

### Binding of human importin-α1, -α5 and -α7 to human-like vRNPs depends on NP

Previous reports highlighted the involvement of PB2 E627K substitution in vRNP complex formation via improved binding of the PB2-627K containing trimeric polymerase to NP [10], [11]. It has been shown that single NLS-containing cargo proteins might be bound and transported into the nucleus by several importin-α isoforms. However, these cargo proteins might differ in their importin-α isoform binding preferences when expressed in complexes or in competition with other cargo proteins for nuclear import. It has been demonstrated that both, the NLS and the protein context are responsible for distinct importin-α binding specificities upon competition of multiple substrates for the limited amount of importin-α proteins [20]. Since importin-α binding to monomeric PB2 did not provide an explanation for the altered polymerase activities in importin-α silenced cells, we next investigated whether binding of importin-α proteins to vRNP complexes was affected.

**Figure 3:**
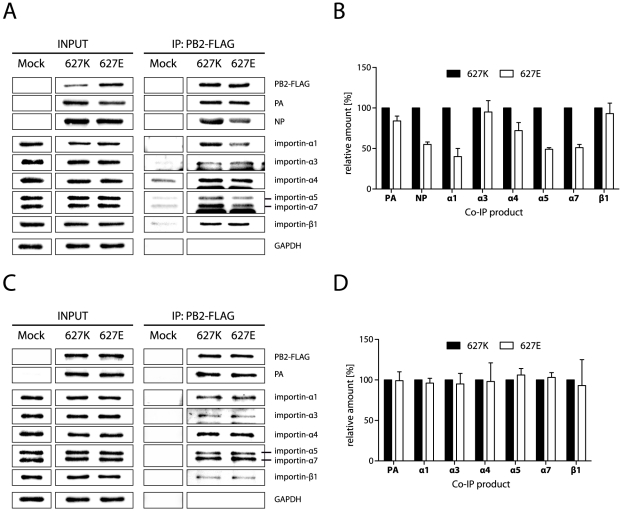
NP mediates increased binding of importins to PB2-627K containing vRNPs. (A) vRNP binding to endogenous importins. 293T cells were co-transfected with plasmids encoding PB2-627K-FLAG or PB2-627E-FLAG, PB1, PA, NP as well as the pPol-I-NP-Luc construct. Mock transfected cells were used a control (Mock). Cells were lysed 48 h after transfection and immunoprecipitation was performed using the FLAG-tag. The amount of co-immunoprecipitated PA, NP, importin-α1, -α3, -α4, -α5, -α7 and -β1 was analyzed by Western blotting. (B) Quantification of vRNP binding to endogenous importins in (A) using a Bioimager as described in Experimental Procedures. Amounts of Co-IP products were normalized against precipitated PB2 levels. Data shown represent the mean +/− SD of at least three independent experiments. (C) Trimeric polymerase binding to endogenous importins. Co-immunoprecipitation was performed as in (A), except NP expressing plasmid was omitted. (D) Quantification of trimeric polymerase binding to endogenous importins in (C) using a Bioimager as described in Experimental Procedures. Amounts of Co-IP products were normalized against precipitated PB2 levels. Data shown derived from four to six independent experiments and represent the mean +/− SD.

Therefore, viral vRNP complexes were reconstituted in 293T cells by co-transfection of expression plasmids for PB2-627K-FLAG or PB2-627E-FLAG, PB1, PA and NP along with pPol-I-NP-Luc to provide an RNA template. vRNP complexes were precipitated from cell lysates using the FLAG-tagged PB2 proteins and the amount of importin-α1, -α3, -α4, -α5, -α7 and -β1 co-immunoprecipitated along with both vRNP complexes was analyzed by Western blotting and quantified (Figure 3A and 3B). vRNP complex formation was confirmed by detection of co-immunoprecipitated PA as well as NP protein. PB2-627K-FLAG or PB2-627E-FLAG containing vRNP complexes precipitated comparable amounts of PA suggesting that PB2 E627K substitution does not affect trimeric polymerase complex formation. However, vRNP complexes containing PB2-627E-FLAG precipitated less NP protein (55%) compared to human-like vRNP complexes. Additionally, we observed a strong reduction in binding to importin-α1 (40%), -α5 (49%) and -α7 (51%). No obvious differences have been observed for importin-α3 and -α4 with both vRNP complexes. Interestingly, similar amounts of the importin-β1 receptor were precipitated (Figure 3B).

Next, co-immunoprecipitation assays were performed with the polymerase complex (PB1, PB2 and PA) omitting NP (Figure 3C and 3D). Remarkably, differential importin-α binding was lost with PB2 627K and PB2 627E polymerase complexes, indicating that NP is involved in importin-α1, -α5 and -α7 binding to human-like polymerase complexes.

**Figure 4:**
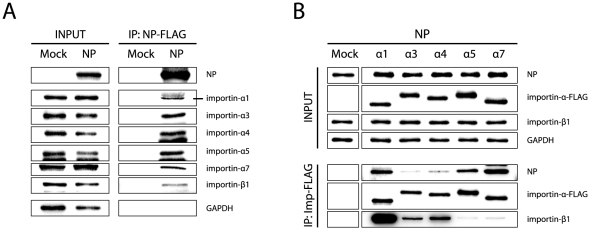
NP binding affinity to individual importin-α isoforms. (A) NP binding to endogenous importins. 293T cells were either Mock transfected or with plasmids encoding NP-FLAG and lysed 48 h after transfection. Immunoprecipitation was performed using the FLAG-tag. Amount of co-immunoprecipitated importin-α1, -α3, -α4, -α5, -α7 and -β1 was determined by Western blot analysis. (B) NP binding to overexpressed importins. 293T cells were co-transfected with plasmids encoding FLAG-tagged importins (α1, α3, α4, α5 or α7) and NP. NP-only transfected cells served as a control (Mock). Cells were lysed 48 h after transfection and immunoprecipitated using the FLAG-tag. The amount of co-immunoprecipitated NP and importin-β1 was determined by Western blot analysis.

To further understand the role of NP in importin-α binding, interaction between NP and endogenous importins was confirmed in 293T cells (Figure 4A). Co-immunoprecipitation assays were performed with importin-α-FLAG and NP proteins expressed in 293T cells (Figure 4B). NP bound to all importins with strongest affinities for importin-α1, -α5 and -α7. Again, importin-β1 showed lowest affinity to importin-α5 and -α7 as observed with monomeric PB2. The overall abundance of importin-α5 and -α7 compared to importin-β1 levels which are not observed with importin-α1, -α3 or –α4 might suggest additive functions for importin-α5 and -α7 beyond nuclear transport.

Our findings demonstrate that PB2 E627K substitution induces binding of importin-α1, -α5 and -α7 proteins to human-like vRNP complexes mediated by NP. Interestingly, importin-β1 binding properties were not changed despite increased importin-α1, -α5 and -α7 binding to PB2 627K human-like vRNPs. Thus, differential importin-α binding observed here with the PB2 627 signature does not correlate with differences in importin-β1 binding which is indicative of altered nuclear transport as previously shown for the PB2 701 adaptive position [4]. Furthermore, highest binding affinity of NP for importin-α1, -α5 and -α7 correlates with the recruitment of the same importin isoforms to human-like vRNPs. This underlines the bridging role of NP in vRNP/importin-α complex formation.

### Silencing of human importin-α1, -α3 and -α7 does not affect subcellular distribution of vRNP subunits PB2 or NP

**Figure 5:**
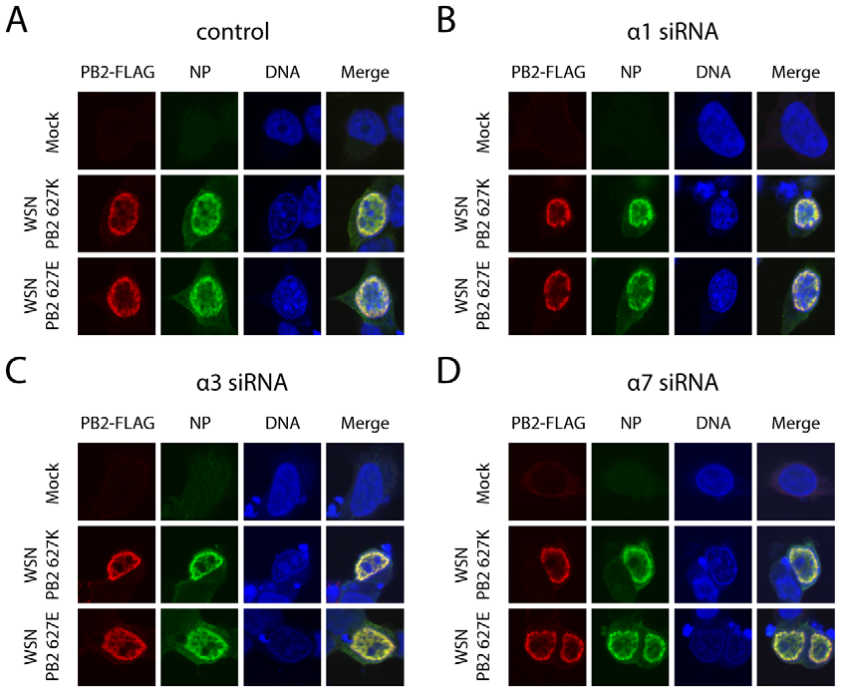
Importin-α silencing does not affect subcellular localization of PB2 or NP. (A–D) vRNP complexes containing either WSN-PB2-627K-FLAG or WSN-PB2-627E-FLAG were expressed in unsilenced controls (A), importin-α1 (B), -α3 (C), or -α7 (D) silenced 293T cells. Subcellular distribution of PB2 and NP was analyzed by specific staining against PB2-FLAG and NP protein. Mock transfected cells were used as negative controls.

PB2 627K containing human-like vRNP/importin-α complexes did not result in increased importin-β1 binding which is indicative of improved nuclear import of vRNP components [4]. To further investigate, whether PB2 E627K substitution affects subcellular distribution of vRNP subunits, we analyzed PB2 and NP localization by immunofluorescence assays in unsilenced as well as importin-α silenced cells (Figure 5). vRNP complexes containing either PB2-627K-FLAG or PB2-627E-FLAG were reconstituted in 293T cells and specific antibodies were used to stain for PB2-FLAG and NP. Here, we focused on the importin-α1, -α3 and -α7 isoforms which significantly altered viral polymerase activity (Figure 1A and B). In unsilenced controls, both PB2 variants as well as NP were detected only in nuclear areas of the cell. No differences were observed in PB2 subcellular localization with either PB2 627K or PB2 627E (Figure 5A). In importin-α silenced cells, localization of PB2 and NP was not affected compared to unsilenced controls (Figure 5B–D).

Taken together, we show that adaptive mutation in PB2 E627K does not affect subcellular distribution of the vRNP subunits PB2 or NP. Further, importin-α1, -α3 and -α7 isoforms are not essentially required for nuclear accumulation of these vRNP subunits.

### Replication of WSN-PB2_627K_ depends on importin-α1 and -α7 in human lung cells

**Figure 6:**
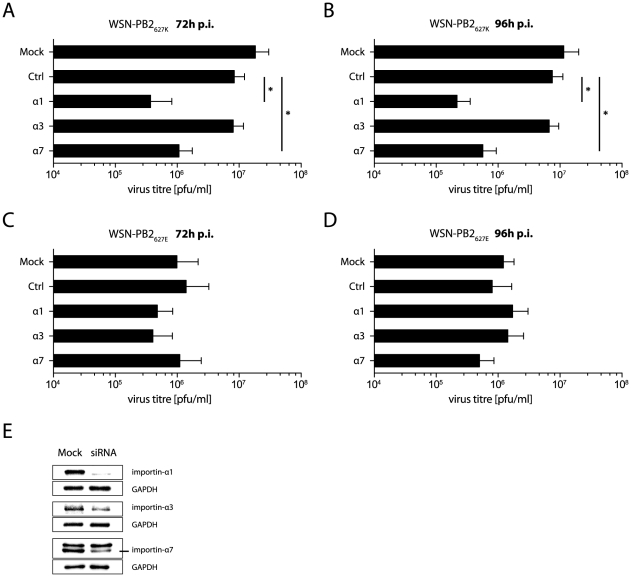
Human- but not avian-like virus growth depends on importin-α1 and -α7. (A–D) Virus growth in importin silenced human cells. A549 cells were silenced using siRNA for importin-α1, -α3 or -α7 and infected at MOI of 0.001 with WSN-PB2_627K_ (A and B) or WSN-PB2_627E_ (C and D). Unsilenced (Mock) and control siRNA transfected cells (Ctrl) were used as a control. Virus titres were determined by plaque assay 72 and 96 hours post infection. As a further control negative siRNA silenced cells were infected (Ctrl). Data shown represent means of three to six independent experiments +/− standard deviations (SD) (*p<0.05, **p<0.01, ***p<0.001, by students *t*-test). (E) Confirmation of importin-α silencing in human A549 cells by Western blot analysis. GAPDH was used as a loading control.

In order to assess whether the regulatory role of importins on polymerase activity affects virus growth, we have performed growth kinetics of recombinant viruses containing either PB2 627K (WSN-PB2_627K_) or PB2 627E (WSN-PB2_627E_) in the WSN (H1N1) virus background in importin-α silenced A549 human lung cells (Figure 6E). Silencing of importin-α1 and -α7 significantly decreased growth of WSN-PB2_627K_ virus by 10- to 50-fold, both 72h (2% and 6%) and 96h p.i. (2% and 5%) compared to controls (Figure 6A and B). Silencing of importin-α3 had no significant effect on WSN-PB2_627K_ replication. WSN-PB2_627E_ growth was not affected in any of the importin silenced cells (Figure 6C and D).

These findings show that human-like PB2 627K but not avian-like PB2 627E virus growth depends on importin-α1 and -α7 in human lung cells. However, an inhibitory effect of importin-α3 on viral replication was not observed.

### Importin-α7^−/−^ mice are less susceptible to WSN-PB2_627K_ infection

**Figure 7:**
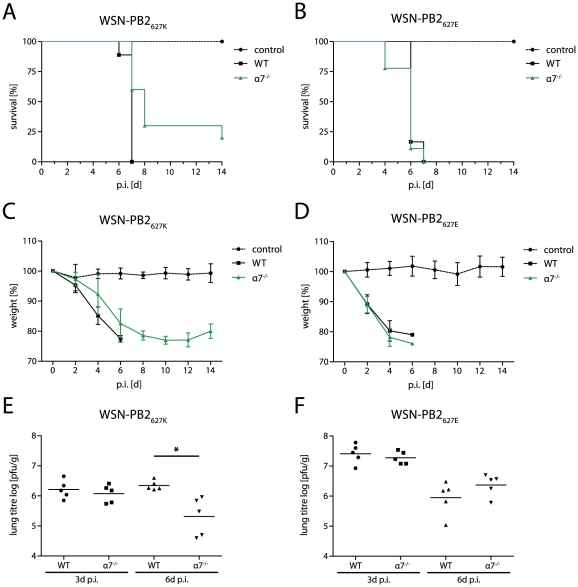
Importin-α7^−/−^ mice are less susceptible to human- but not avian-like virus infection. (A–F) Pathogenicity of human- and avian-like virus in wildtype and importin-α7^−/−^ mice. Wildtype (*n* = 16) or importin-α7^−/−^ (*n* = 16) mice were infected with 10^5^ p.f.u. (∼20-fold MLD_50_) of WSN-PB2_627K_ (A, C and E) or 5×10^6^ p.f.u. (∼10-fold MLD_50_) of WSN-PB2_627E_ (B, D and F). Survival (A and B) and weight loss (C and D) were monitored for 14 days. Mice receiving PBS were used as controls. Data shown representative average values per group. (E and F) Lungs were removed on days 3 (*n* = 5) and 6 (*n* = 5) post infection (p.i.). Virus titres were determined by plaque assay.

Next, we wanted to study whether the positive regulatory role of importin-α7 on human- but not avian-like polymerase activity is also reflected *in vivo*. It has been shown that a single amino acid substitution in PB2 E627K converts a non-lethal virus to a lethal virus in mice [9]. Therefore, we have determined the MLD_50_ of WSN-PB2_627K_ and WSN-PB2_627E_ viruses using serial virus dilutions (Table S1). We chose the minimum inoculation dose for each virus which guarantees 100% lethality in WT mice. This would allow the detection of potential differences in importin-α7^−/−^ mice in case the *in vitro* data should also correlate *in vivo*. Therefore, we have infected wildtype and importin-α7^−/−^ mice with 10^5^ p.f.u. (∼20-fold LD_50_) of WSN-PB2_627K_ or 5×10^6^ p.f.u. (∼10-fold LD_50_) of WSN-PB2_627E_. Wildtype animals infected with WSN-PB2_627K_ displayed significant weight loss and succumbed to infection within 7 days (Figure 7A and C). In contrast, importin-α7^−/−^ mice lost less weight and 20% of the infected animals survived an otherwise 100% lethal infection. No significant differences in weight loss or survival were observed among importin-α7^−/−^ and wildtype mice infected with WSN-PB2_627E_ (Figure 7B and D). All mice died within 7 days. Reduced mortality of WSN-PB2_627K_ infected importin-α7^−/−^ mice correlated with 10-fold reduction in lung titers, reduced expression of virus antigens in the lung and inflammatory cells on day 6 p.i. but not on day 3 p.i. compared to WT animals (Figure 7E, Figure S7). This might suggest that initial replication deficiencies are detected at later time points upon viral clearance by the host similar to previous observations in importin-α7^−/−^ mice [5]. In contrast, no significant differences in lung titres, virus antigen positive cells or lung pathology were observed in WSN-PB2_627E_ infected WT and importin-α7^−/−^ mice on day 3 and 6 p.i. (Figure 7F and Figure S7).

Furthermore, serial viral dilution experiments (Table S1 and Figure S8) confirmed the significant effect of importin-α7 on WSN-PB2_627K_ but not WSN-PB2_627E_ infection. Survival further increased in importin-α7^−/−^ mice infected with 10^4^ p.f.u. of WSN-PB2_627K_ up to 60% compared to 70% lethality in WT mice. In contrast, no significant differences were observed upon infection with WSN-PB2_627E_ between WT and importin-α7^−/−^ mice.

In summary, these data demonstrate that human-like PB2 627K but not avian-like PB2 627E confers importin-α7 dependency in mice.

## Discussion

The influenza virus polymerase complex is active in the nucleus of the infected cell where cytoplasmically expressed PB1-PA dimers are imported by RanBP5 [21]–[23], a component of the non-classical import machinery which is independent of importin-α. PB2 appears to be imported separately into the nucleus by the classical importin-α/β1 mediated import pathway [4], [15]. Several mammalian influenza virus strains either having PB2 701N, 627K or other unknown adaptive positions in 2009 pandemic influenza virus strains displayed importin-α7 dependency in human lung cells [5]. PB2 D701N and NP N319K were sufficient to confer importin-α7 dependency in human cells. However, the role of other host adaptive positions, such as PB2 627K was not further investigated. PB2 627K can be functionally substituted by PB2 701N in terms of polymerase activity [24] and virus transmission [19], [24]. These findings suggested a mechanistically similar mode of action between PB2 627 and PB2 701. Therefore, we investigated whether the cellular importin-α proteins are also involved in PB2 E627K mediated host adaptation and pathogenicity as has been shown before for PB2 701N [4], [5].

Two opposing concepts have been proposed on the involvement of cellular factors concerning the PB2 627 signature position: (1) Mehle and Doudna hypothesized that an inhibitory factor exists which restricts avian-like polymerase activity in mammalian but not in avian cells [11], (2) Moncorgé and colleagues suggested that there is no inhibitory factor of avian polymerases but the absence or low expression of a positive factor is responsible for their low polymerase activity in human cells [12]. Indeed, very recently Bortz et al. have identified several cellular proteins which differentially regulate H5N1 polymerase activity in a PB2 627 dependent fashion [14]. Among them, the cellular DDX17 protein was described as a novel factor to facilitate efficient human-adapted PB2 627K H5N1 virus transcription and replication in human cells. Future investigation is needed to analyze the role of these host factors [14] within the network of polymerase interacting proteins [13] in host adaptation and pathogenicity in relevant animal models.

In this study, we could identify the importin-α isoforms as novel regulators of PB2 627K mediated human-like polymerase activity and pathogenicity. Human importin-α1 and -α7 act as positive regulators of PB2 627K mediated human-like polymerase activity while importin-α3 is a negative regulator independently of the viral polymerase origin. vRNP expression levels were not affected in importin-α silenced cells (Figure S1). Thus, it is unlikely that these effects are due to altered expression of vRNP subunits. We have performed all experiments in this study in the WSN (H1N1) background for consistency with previous studies on PB2 627 mediated polymerase activity and vRNP formation in human cells [11], [25]. Thus, our findings with importin-α1 and -α7 as positive regulators of human- but not avian-like polymerase activity are consistent with the model suggested by Moncorgé and colleagues since reduced avian-like polymerase activity correlated with reduced binding of these importin-α isoforms in human cells. However, this does not exclude the existence of another inhibitory factor in human cells since host adaptation is a multigenic process and importins are one of the factors involved in host adaptation and pathogenesis.

In contrast to PB2 D701N, PB2 E627K substitution did not affect importin-α binding affinity when expressed alone. This is consistent with previous reports using the C-terminal region of PB2 only [16]. The authors showed that PB2 D701N increases importin-α binding in general, consistent with previous studies [4], [5]. In contrast, PB2 E627K substitution did not alter importin-α binding properties [16] consistent with our data described here. However, single cargo proteins can interact with most of the importin-α isoforms while expression of multiple cargos can result in distinct importin-α specificities which is determined not only by the NLS but the whole protein context [20]. In case of a viral infection, multiple viral and cellular proteins compete for nuclear entry by the importin-α/β1 pathway. Limited availability of the importin-α isoforms is believed to be a restricting factor.

Therefore, we have further analyzed whether the expression of vRNPs affects binding to individual importin-α isoforms in a PB2 627 dependent manner. While PB2 627K alone had no effect on importin-α binding, it enhanced binding of importin-α1, -α5 and -α7 to vRNPs when expressed with all vRNP subunits. The enhanced binding of importin-α1 and -α7 to human- but not avian-like vRNPs correlated with their positive regulatory effect on human-like but not avian-like polymerase activity. However, reduced binding of importin-α1, -α5 and -α7 to avian-like vRNPs did not correlate with reduced precipitation of importin-β1 from vRNP/importin-α which is indicative for altered nuclear transport [4]. Consistently, subcellular localization of PB2 627K or NP was not affected unlike PB2 701N and NP 319K [4]. This is further confirmed by studies performed here and by others using cell fractionation assays where PB2 E627K did not affect subcellular distribution upon polymerase transfection or virus infection (Figure S4) [11].

According to the literature, cargo proteins are transported into the nucleus as cargo/importin-α/β1 ternary complexes [17], [18]. Therefore, one would expect similar importin-β1 proteins bound to PB2/importin-α complexes. However, it is still unclear how many importin-α isoforms are attached to ternary complexes to mediate efficient nuclear transport. The abundance of importin-α5 and -α7 compared to importin-β1 levels might either suggest that several importin-α proteins are associated with ternary complexes or, more likely that these isoforms have additional functions to those of nuclear transport. Indeed, it has been postulated before that importins can fulfill multiple functions. Importin-β-like proteins were shown to act as chaperons for exposed basic domains [26]. Very recently, importin-α/β1 proteins were suggested to play a role in cellular mRNA quality control [27]. The idea that importin-α isoforms might have additional functions than nuclear transport in the influenza virus life cycle was first put forward by Resa Infante et al. They proposed that host-dependent interaction of importin-α with PB2 is required for virus RNA replication itself [28]. Future investigation is needed to understand whether this function is affected by the PB2 627K signature position.

Another possibility how importin-α might regulate human-like polymerase activity would be indirectly by recruiting other cellular factors to their vRNPs by specific binding to importin-α1 and -α7. However, silencing both, importin-α1 and -α7 did not further suppress PB2 627K polymerase activity in an additive manner (Figure S2). This might suggest that importin-α1 and -α7 have similar functions and that other cellular factors are involved which are not specifically bound and transported by these isoforms. Further analyses are needed to identify importin-α specific and unspecific host cell factors within the polymerase interacting network and their contribution on host dependent polymerase activity and pathogenicity. Other groups have further postulated based on structural and biological data that importin-α proteins might play a role in polymerase assembly [15], [16], [29]. PB2 E627K was shown to modulate vRNP complex formation in mammalian cells [10] consistent with our findings here. Our studies suggest that enhanced binding of importin-α1, -α5 and -α7 to human-like vRNPs is mediated by NP. This is further supported by the fact that the same importins show highest affinity for NP when expressed alone, in the vRNP context or in infection (Figure S3). However, our studies cannot distinguish whether enhanced importin-α binding to human-like vRNPs is due to defects in assembly or a subsequent consequence that less efficiently NP is bound to the polymerase complex. Purified human-like vRNPs from importin-α silenced cells did not show altered PB2/NP ratios (Figure S5) suggesting that differential importin-α binding to human-like vRNPs is likely to present a consequence than a cause of defective vRNP assembly. However, we cannot exclude from these studies the role of importins in polymerase or vRNP formation. Clearly, future investigation is needed to distinguish between all these possibilities and whether these functions are regulated by host adaptive signatures. It would also be interesting to analyze whether the PB2 D701N substitution also affects transport independent functions mediated by importins since the functional substitution described for PB2 627K and 701N [19] might suggest alternative transport independent pathways which are affected by both mammalian signatures.

The differential role of importins was further confirmed with recombinant virus containing human-like PB2 627K which displayed dependency on importin-α1 and -α7 in human lung cells. Interestingly, importin dependency was lost with avian-like PB2 627E substitution. However, the general inhibitory activity of importin-α3 was restricted to vRNPs and was not observed in the viral context, since silencing of importin-α3 did not affect human- or avian-like virus growth kinetics in human lung cells. Specificity to individual importin-α isoforms is likely due to competition of multiple cellular and viral proteins for nuclear import [5], [20]. Upon infection, the restricting activity of importin-α3 for vRNPs might be overcome by another viral factor besides the polymerase complex. Previously, dependency on importin-α3 was mainly observed with HPAIV. However, the role of importin-α3 on polymerase activity of HPAIV was not studied [5]. Clearly, further studies are needed to identify the factor responsible for overcoming the restricting activity of importin-α3 observed with the mammalian influenza virus strain used in this study.

Remarkably, the regulatory role of importin-α7 observed *in vitro* could also be verified *in vivo*. Importin-α7^−/−^ mice were less susceptible to human- but not avian-like virus infection (Table S1, Figure S8). Accordingly, systemic spread was mainly restricted to the lung in human-like influenza virus infected importin-α7^−/−^ mice compared to WT mice where infection of the brain was observed (Figure S6 and S7). In contrast, no difference in survival, virus titres or lung pathology has been observed with avian-like virus in wildtype or importin-α7^−/−^ mice (Table S1, Figure S6, S7 and S8). Surprisingly, viremia has been observed for human- and avian-like viruses (Figure S6). However, this did not necessarily lead to virus replication in the brain with the avian-like virus suggesting that PB2 627K is needed for efficient systemic spread. This further supports that PB2 627K and the importin-α7 gene are important for efficient systemic spread and extrapulmonary infection of human-like influenza viruses.

In summary, these findings strongly suggest that importin-α1 and -α7 isoforms play an important role in host adaptation of several mammalian viruses with either PB2 627K or PB2 701N adaptive positions [5]. However, the primary role of importin-α1 and -α7 in nuclear transport was not pivotal for PB2 E627K mediated host adaptation suggesting that importins are multifunctional proteins modulating viral polymerase activity by novel but yet unknown mechanisms. Further, our observations highlight the impact of importin-α isoforms in interspecies transmission of influenza viruses. Therefore, targeting especially importin-α7 may provide a strategy with therapeutic potential against human influenza viruses.

## Materials and Methods

### Ethics statement

Animal experiments were performed according to the guidelines of the German animal protection law. All animal protocols were approved by the relevant German authority (Behörde für Stadtentwicklung und Umwelt Hamburg). Mice were humanely killed upon ≥25% weight loss according to the guidelines of the German animal protection law.

### Animal experiments

Importin-α7^−/−^ mice [5], [30] and wildtype littermates in the C57BL/6J genetic background were bred and housed at the animal facility of the Heinrich-Pette-Institute, Leibniz Institute of Experimental Virology in Hamburg, Germany. Wildtype (*n* = 20) and importin-α7^−/−^ (*n* = 20) mice were intranasally infected with 10^5^ p.f.u. (∼20-fold MLD_50_) of WSN-PB2_627K_ or 5×10^6^ p.f.u. (∼10-fold MLD_50_) of WSN-PB2_627E_. Survival and weight loss was monitored for 14 days. Five animals were sacrificed on day 3 and 6 post infection (p.i.). Lungs and brains were removed and blood was obtained by heart-punctuation. Viral titers were determined by plaque assay.

MLD_50_ studies were performed using serial dilutions of WSN-PB2_627K_ (10^3^, 10^4^ and 10^5^ p.f.u.) or WSN-PB2_627E_ (10^5^, 10^6^ and 5x10^6^ p.f.u.) in wildtype and importin-α7^−/−^ mice using additional mice (*n* = 5–10) per group and per dose.

### Cells and viruses

Cell lines of human embryonic kidney cells (293T), human alveolar adenocarcinoma cells (A549) and chicken fibroblasts (DF1) were grown in DMEM (Dulbecco's modified Eagle's medium, PAA) supplemented with 10% fetal calf serum (PAA), 1% penicillin/streptomycin (PAA) and 1% L-Glutamine (PAA) at 5% CO_2_ and 37°C. Recombinant A/WSN/33 viruses WSN-PB2_627K_ and WSN-PB2_627E_ were rescued using the pHW2000 based 8-plasmid system as described previously [31].

### Transfection and vectors

All transfections were performed using Lipofectamine 2000 (Invitrogen) according to the manufacturer's instructions. Vector constructs used were pHW2000-(WSN-PB2-627K, WSN-PB1, WSN-PA, WSN-NP, WSN-HA, WSN-NA, WSN-M, WSN-NS) kindly provided by H.-D. Klenk (Institute of Virology, Marburg, Germany), pHW2000-(WSN-PB2-627K-FLAG, WSN-PB2-627E, WSN-PB2-627E-FLAG, NP-FLAG), pPol-I-NP-Luc-human [3], pPol-I-NP-Luc-chicken (kindly provided by M. Schwemmle, Institute of Virology, Freiburg, Germany), pRL-TK (PROMEGA), pcDNA-importin-α1-FLAG, pcDNA-importin-α3-FLAG, pcDNA-importin-α4-FLAG, pcDNA-importin-α5-FLAG and pcDNA-importin-α7-FLAG. The pcDNA-importin-α-FLAG constructs were described previously [5]. Constructs pHW2000-WSN-PB2-627K-FLAG and pHW2000-WSN-NP-FLAG constructs were generated by standard PCR techniques attaching a FLAG M2 tag to the C-terminus of the coding sequence. pHW2000-WSN-PB2-627E and pHW2000-WSN-PB2-627E-FLAG constructs were generated by site-directed mutagenesis.

### Antibodies

Primary antibodies used for Western blot analysis, cell fractionation and immunofluorescence assays include mouse anti-FLAG (Sigma), rabbit anti-importin-α1 (Abcam), goat anti-importin-α3 (Abcam), goat anti-importin-α4 (Abcam), rabbit anti-importin-α5/α7 (kindly provided by E. Hartmann, Institute of Biology, Lübeck, Germany), mouse anti-importin-β1 (BD Transduction Laboratories), rabbit anti-GAPDH and rabbit anti-LSD1 (Cell signaling), mouse anti-PB2 (kindly provided by J. Ortín, CSIC, Madrid, Spain), rabbit anti-PA (kindly provided by G.G. Brownlee and E. Fodor, University of Oxford, United Kingdom), rabbit anti-FPV-serum [4], rabbit anti-NP (Abcam). Secondary HRP-conjugated antibodies used were anti-mouse-HRP, anti-rabbit-HRP and anti-goat-HRP (Sigma). For immunofluorescence, donkey anti-mouse-Cy3 and donkey anti-rabbit-Cy2 secondary antibodies were obtained from Jackson ImmunoResearch.

### Polymerase activity assay

293T cells were transfected with siRNA designed against human importins (α1, α3, α4, α5 and α7) as described previously [5]. Allstars negative control siRNA (QIAGEN) was used as a control. 48h after silencing, cells were transfected with pHW2000 vector constructs encoding PB2-627K or PB2-627E, PB1, PA and NP with reporter constructs pPol-I-NP-Luc encoding firefly and for normalization pRL-TK (Promega) for renilla luciferase. Luciferase activity was measured 24 h after transfection according to the manufacturer's instructions. The vRNP reconstitution assay has been performed according to previously validated experimental settings described before [3]. Successful siRNA mediated silencing of human importin-α isoforms was confirmed using Western blot analysis.

### Co-immunoprecipitation assay

All immunoprecipitations were performed using EZview Red ANTI-FLAG M2 affinity gel (Sigma) and eluted using a 3x FLAG peptide (Sigma) according to the manufacturer's instructions. As expression levels of PB2 627K or 627E can vary in the vRNP context [10], we have adjusted co-precipitated PB2 levels when expressed as vRNPs by serial dilution. Quantification of co-immunoprecipitation products was performed with the Bioimager Image Quant LAS 4000 at non-saturated levels. Relative amounts of co-immunoprecipitated products associated with PB2, vRNPs or trimeric polymerase complexes (PB1, PB2 and PA) containing PB2 627K were set to 100%.

### Subcellular fractionation assay

Experimental setting was performed as described before [4]. Briefly, 293T cells were infected with an MOI of 2 and subcellular fractions were analyzed 6h p.i. Nuclear and cytoplasmic fractions were obtained using the NE-PER Kit (Pierce) according to the manufactures instructions. Protein concentration of each fraction was measured by BCA Protein Assay (Pierce). Subcellular distribution of viral and cellular proteins was analyzed by Western blot analysis. Equal amount of protein was loaded for each fraction. Fractionation purity was controlled using specific subcellular markers, such as GAPDH as a cytoplasmic protein and LSD1 as a nuclear protein.

### Immunofluorescence assay

293T cells were silenced for importin-α1, -α3 or -α7 using siRNA as described before [5]. Cells were co-transfected with plasmids encoding PB2-627K-FLAG or PB2-627E-FLAG, PB1, PA, NP as well as with pPol-I-NP-Luc 48h after siRNA transfection and seeded on glass cover slips for 24h. Cells were fixed with 3% paraformaldehyde in PBS for 10 min at room temperature and permeabilized with methanol for 5 min at −20°C. Permeabilized cells were blocked with 5% donkey serum (Abcam) in PBS for 1 h and stained with antibodies directed against PB2-FLAG and NP. Cellular DNA was stained with DRAQ5 (Cell signaling). All images were taken on a confocal laser scanning microscope (Zeiss 510 Meta CLSM) in multitrack mode with x63/1.4 oil Plan-Apochromat objective. Zeiss Confocal Microscopy Software (Release 3.29) was used.

### Analysis of virus growth

A549 cells were silenced for importin-α1, -α3 or -α7 using siRNA as previously described [5] and infected with recombinant A/WSN/33 virus containing either PB2 627K or PB2 627E at MOI 0.001. Allstars negative control siRNA (QIAGEN) was used as a control. Supernatants were taken 72 hours and 96 hours post infection (p.i.). Virus titers were determined as plaque forming units (p.f.u.) by plaque assay as described before [5].

### Immunohistochemistry

For histopathological examination, lungs of infected wildtype and importin-α7^−/−^ mice were sectioned on day 6 p.i. and treated as described previously [32]. Viral antigens were stained using anti-FPV-serum and ZytoChem Plus HRP-DAB Kit Broad Spectrum (Zytomed) according to the manufacturer's instructions.

### Statistics

For statistical analysis of experimental data, mean values +/− standard deviation (SD) were calculated and *p*-values were obtained according to student's *t*-test for paired data. For comparison of animal survival rates Geham-Brelow-Wilcoxon test was used. Statistical significance was defined as *p*<0.05 (*p<0.05, **p<0.01, ***p<0.001).

## Supporting Information

Figure S1 **Importin-α silencing does not alter PB2 or NP expression levels.** WSN-PB2 627K or WSN-PB2 627E containing vRNPs were reconstituted in unsilenced controls or in importin-α1, -α3 or -α7 silenced 293T cells. Cells were lysed 24 h after transfection and expression levels of PB2 and NP proteins were analyzed by Western blotting. GAPDH was used as a loading control.
(EPS)

Figure S2 **Combinational silencing of importin-α1 and -α7 does not result in additive effects.** Polymerase activity of WSN-PB2-627K containing vRNPs in importin-α1 and -α7 silenced 293T cells. Activity of vRNPs in negative siRNA controls (Ctrl) was set 100%. As a further control, vRNPs were transfected omitting the PB2 subunit in negative siRNA silenced cells (Ctrl). Means of at least three independent experiments +/− standard deviations (SD) are shown (*p<0.05, **p<0.01, ***p<0.001, by students *t*-test).
(EPS)

Figure S3 **Importin-α binding affinities of NP upon infection are comparable to monomeric NP.** NP binding to overexpressed importins during infection. 293T cells were co-transfected with plasmids encoding FLAG-tagged importins (α1, α3, α4, α5 or α7) or Mock transfected (Mock). Cells were infected at MOI of 2 with WSN-PB2_627K_ (A) or WSN-PB2_627E_ (B). Cells were lysed 8h p.i. and immunoprecipitated using the FLAG-tag. The amount of co-immunoprecipitated NP and importin-β1 was determined by Western blot analysis.
(EPS)

Figure S4 **PB2 E627K does not alter subcellular localization of PB2 or NP upon infection.** 293T cells were infected with an MOI of 2. Subcellular fractions were analyzed at 6h p.i. Western blotting was performed using equal protein amounts of each fraction. Fraction purity was controlled using GAPDH as a cytoplasmic marker and LSD1 as a nuclear marker.
(EPS)

Figure S5 **Importin-α silencing does not affect WSN-PB2 627K mediated vRNP complex formation.** vRNP complex formation after importin-α silencing. 293T cells were transfected with siRNA targeted against importin-α1, -α3 or -α7 or negative control siRNA (Ctrl). 48 hours after siRNA treatment, cells were co-transfected with plasmids encoding PB2-627K-FLAG, PB1, PA, NP as well as the pPol-I-NP-Luc construct. Mock transfected cells served as a control (Mock). Cells were lysed 24h after transfection and importin-α silencing was confirmed by Western blot analysis. Lysates were subjected to immunoprecipitation using the FLAG-tag and the amount of co-immunoprecipitated PA and NP was analyzed by Western blotting.
(EPS)

Figure S6 **PB2 627K and importin-α7 are crucial for systemic spread in mice.** Wildtype (*n* = 5) or importin-α7^−/−^ (*n* = 5) mice were infected with either 10^5^ p.f.u. (30-fold MLD_50_) of WSN-PB2_627K_ (A and C) or 5×10^6^ p.f.u. (B and D) (10-fold MLD_50_) of WSN-PB2_627E_. Virus titres were determined in blood and brain at days 3 and 6 p.i. by plaque assay. Titers below the detection limit of the plaque assay (3 p.f.u.) could not be detected. Mice receiving PBS were used as controls.
(EPS)

Figure S7 **Human-like virus presents reduced replication and pathology in importin-α7^−/−^ mice.** Lung pathology of wildtype (*n* = 5) (B and D) or importin-α7^−/−^ (*n* = 5) (C and E) mice infected with either 10^5^ p.f.u. (30-fold MLD_50_) of WSN-PB2_627K_ or 5×10^6^ p.f.u. (10-fold MLD_50_) of WSN-PB2_627E_. Viral antigen expression was analyzed on day 6 p.i. by immunohistochemical staining as described in Experimental Procedures. Mice receiving PBS were used as controls (A).
(TIF)

Figure S8 **Importin-α7^−/−^ mice show reduced mortality upon human-like but not avian-like virus infection compared to WT mice.** (A–B) Mortality of human- and avian-like virus in wildtype and importin-α7^−/−^ mice. Wildtype or importin-α7^−/−^ mice were infected with 10^4^ p.f.u. of WSN-PB2_627K_ (A) or 10^6^ p.f.u. of WSN-PB2_627E_ (B) as described in Materials and Methods. Survival was monitored for 14 days. Mice receiving PBS were used as controls.
(EPS)

Table S1 **Virulence of WSN-PB2_627K_ and WSN-PB2_627E_ viruses in WT and α7^−/−^mice.** Significance of differences between WT and importin-α7 knockout mice were calculated by Geham-Breslow-Wilcoxon Test on the Kaplan-Meier survival data (*p<0.05 and **p<0.01). Mouse-lethal-dose-50 (MLD_50_) was calculated as described by Reed & Muench [33].
(DOC)
