# Supplementary figures and images for: Human-like PB2 627K Influenza Virus Polymerase Activity Is Regulated by Importin-α1 and -α7
Source: PLoS Pathog. 2012 Jan 19;8(1):e1002488. doi: 10.1371/journal.ppat.1002488 (PMC3262014; doi:10.1371/journal.ppat.1002488)

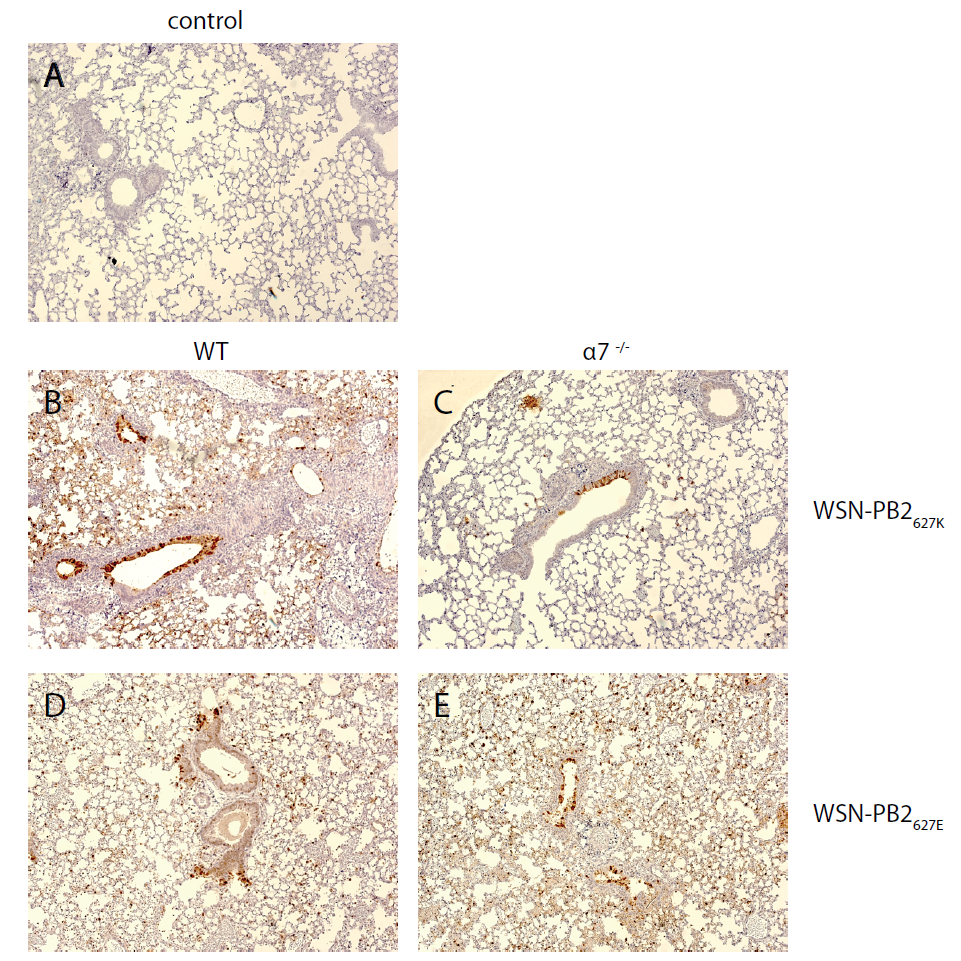

Supplement: Figure S7 — Human-like virus presents reduced replication and pathology in importin-α7−/− mice. Lung pathology of wildtype (n = 5) (B and D) or importin-α7−/− (n = 5) (C and E) mice infected with either 105 p.f.u. (30-fold MLD50) of WSN-PB2627K or 5×10^6^ p.f.u. (10-fold MLD50) of WSN-PB2627E. Viral antigen expression was analyzed on day 6 p.i. by immunohistochemical staining as described in Experimental Procedures. Mice receiving PBS were used as controls (A). (TIF) [file ppat.1002488.s007.tif]
