# Supplementary material for: Human-like PB2 627K Influenza Virus Polymerase Activity Is Regulated by Importin-α1 and -α7
Source: PLoS Pathog. 2012 Jan 19;8(1):e1002488. doi: 10.1371/journal.ppat.1002488 (PMC3262014; doi:10.1371/journal.ppat.1002488)
Supplement: Table S1 — Virulence of WSN-PB2627K and WSN-PB2627E viruses in WT and α7−/−mice. Significance of differences between WT and importin-α7 knockout mice were calculated by Geham-Breslow-Wilcoxon Test on the Kaplan-Meier survival data (*p<0.05 and **p<0.01). Mouse-lethal-dose-50 (MLD50) was calculated as described by Reed & Muench [33]. (DOC) [file ppat.1002488.s009.doc]

**Supplemental Table**

| **Virus** | **Inoculation dose (p.f.u.)** | **Survival (%)** | | **Significance of survival rates between**  **WT and α7^-/-^** | **MLD_50_ (p.f.u.)** | |
| --- | --- | --- | --- | --- | --- | --- |
| WSN-PB2_627K_ |  | **WT** | **α7^-/-^** |  | **WT** | **α7^-/-^** |
|  | 10^5^ | 0 | 20 | *p*=0.0051** | 10^3.7^ | 10^4.3^ |
|  | 10^4^ | 30 | 60 | *p*=0.0449* |  |  |
|  | 10^3^ | 100 | 100 | *p*=1 |  |  |
|  | 5x10^6^ | 0 | 0 | *p*=0.2095 | 10^5.6^ | 10^5.6^ |
| WSN-PB2_627E_ | 10^6^ | 20 | 20 | *p*=0.3695 |  |  |
|  | 10^5^ | 100 | 100 | *p*=1 |  |  |

**Table S1**
